# Comparative diagnostic performance and stability of deep learning- and CFD-based CT-FFR across vessels, cardiac phases, and centers

**DOI:** 10.3389/fcvm.2026.1883012

**Published:** 2026-07-16

**Authors:** Bin Zhou, Yang Guo, Dongchuang Guo, Su Qian, Zhezhe Huang, Yangfan Zhang, Yifeng Zheng, Zhen Wang, Dong Liu

**Affiliations:** 1 Department of Radiology, Huzhou Central Hospital, Fifth School of Clinical Medicine of Zhejiang Chinese Medical University, Huzhou, Zhejiang, China; 2 Department of Radiology, Affiliated Huzhou Hospital, Zhejiang University School of Medicine, Huzhou, Zhejiang, China; 3 Department of Radiology, Huzhou Central Hospital, Affiliated Central Hospital Huzhou University, Huzhou, Zhejiang, China; 4 Department of Orthopedics, South Taihu Hospital Affiliated to Huzhou College, Huzhou, China; 5 Department of Radiology, Affiliated Hangzhou First People's Hospital, School of Medicine, Westlake University, Hangzhou, China; 6 Zhejiang Key Laboratory of Zero Magnetic Medicine, Affiliated Hangzhou First People's Hospital, School of Medicine, Westlake University, Hangzhou, China

**Keywords:** computed tomography angiography, coronary artery disease, deep learning, diagnostic performance, fractional flow reserve

## Abstract

**Background:**

Although CT-derived FFR (CT-FFR) based on deep learning (DL) and computational fluid dynamics (CFD) is increasingly used for functional ischemia assessment, direct head-to-head multi-center evidence regarding their diagnostic stability in the same cohort remains limited. This study aimed to compare the diagnostic performance and robustness of DL-based versus CFD-based CT-FFR against invasive FFR across coronary branches, cardiac phases, clinical centers, and ischemia-positive gray-zone lesions.

**Methods:**

We retrospectively analyzed 220 patients (277 vessels) who underwent coronary CTA and invasive FFR from two centers. CT-FFR was calculated using representative commercial DL-based and CFD-based algorithms. Diagnostic performance was evaluated using invasive FFR as the reference standard. Subgroup analyses were performed for target vessels, reconstruction phases, imaging centers, and gray-zone lesions.

**Results:**

DL and CFD showed high and similar diagnostic performance. The AUC was 0.90 (95% CI: 0.88-0.93) for DL and 0.89 (95% CI: 0.86-0.92) for CFD, and the difference was not significant (*p* > 0.05). Both methods were strongly correlated with invasive FFR (rho = 0.71 for DL; rho = 0.68 for CFD; both *p* < 0.001). The subgroup analyses showed stable performance across vessels, cardiac phases, and centers (all *p* > 0.05). In gray-zone lesions, DL and CFD showed comparable correct classification rates (86.4% vs. 84.6%, *p* = 0.690) and false-negative rates (13.6% vs. 15.4%, *p* = 0.690).

**Conclusion:**

DL-based and CFD-based CT-FFR showed similar and strong diagnostic performance for detecting hemodynamically significant stenosis. These findings support the potential use of both approaches as non-invasive functional assessment tools in selected patients.

## Introduction

Coronary artery disease (CAD) remains a leading cause of morbidity and mortality worldwide (1). In clinical practice, accurate evaluation of coronary lesions requires both anatomical and functional information. Coronary computed tomography angiography (CCTA) provides a non-invasive assessment of coronary anatomy, but the anatomical degree of stenosis does not always reflect its functional significance. This mismatch may lead to overestimation of lesion severity and unnecessary invasive coronary angiography (ICA) or revascularization in some patients (2, 3). Although invasive fractional flow reserve (FFR) is regarded as the reference standard for identifying ischemia-producing lesions (4, 5), its routine use is limited by its invasive nature, procedural cost, and potential risks.

CT-derived fractional flow reserve (CT-FFR) was developed to bridge the gap between anatomical imaging and functional assessment (6). Early and subsequent multicenter studies demonstrated that CT-FFR improves the discrimination of ischemia-producing coronary stenosis compared with anatomic CCTA alone, using invasive FFR as the reference standard (4–6). Conventional CT-FFR methods mainly rely on computational fluid dynamics (CFD), in which patient-specific coronary anatomy reconstructed from CCTA is combined with physiologic assumptions regarding coronary flow, pressure, and microvascular resistance to estimate pressure loss across stenoses (7–9). These approaches have favorable diagnostic accuracy, but broader clinical implementation may be limited by computational burden, processing time, and dependence on segmentation quality.

More recently, artificial intelligence and deep learning (DL)-based CT-FFR solutions have been developed to automate coronary segmentation, lumen extraction, feature learning, and rapid on-site FFR estimation (10–14). Fully automated CT-FFR platforms may shorten processing time, reduce operator dependence, and increase the technical success rate in routine clinical workflows (6, 10). Recent work directly comparing AI-based and CFD-based FFR software in intermediate-grade stenosis further supports the clinical relevance of evaluating these computational strategies side by side (15). Nevertheless, the technical principles, training references, and physiologic assumptions vary across vendors and algorithms, and direct comparison between DL-based and CFD-based CT-FFR in the same clinical cohort remains relatively limited. Recent status updates also emphasize that CT-FFR adoption differs across regions and vendors, underscoring the need for evidence across multiple platforms and practice settings (16).

Several unresolved issues remain important for clinical translation. First, CT-FFR performance can be less stable near the invasive FFR threshold, particularly in the clinical gray zone around 0.75-0.80, where small errors in lumen segmentation, boundary conditions, image quality, or model estimation can change binary classification (5, 17, 18). Second, CT-FFR robustness across coronary vessels, systolic versus diastolic reconstruction phases, and different imaging centers has not been fully characterized. Third, although many studies have validated individual CT-FFR algorithms, fewer studies have tested whether different computational strategies provide comparable diagnostic information under the same imaging and reference-standard conditions.

Therefore, the novelty of the present multi-center study is the direct head-to-head comparison of representative DL-based and CFD-based CT-FFR algorithms in the same patient and vessel cohort with invasive FFR as the reference standard. Beyond overall diagnostic accuracy, we specifically evaluated algorithm stability across coronary branches, cardiac phases, clinical centers, and ischemia-positive gray-zone lesions. This design was intended to clarify whether the two computational strategies provide consistent functional information in clinically relevant subgroups and to identify practical factors that should be considered when CT-FFR is used in routine CCTA-based decision-making.

## Methods

### Patient population

**Figure 1:**
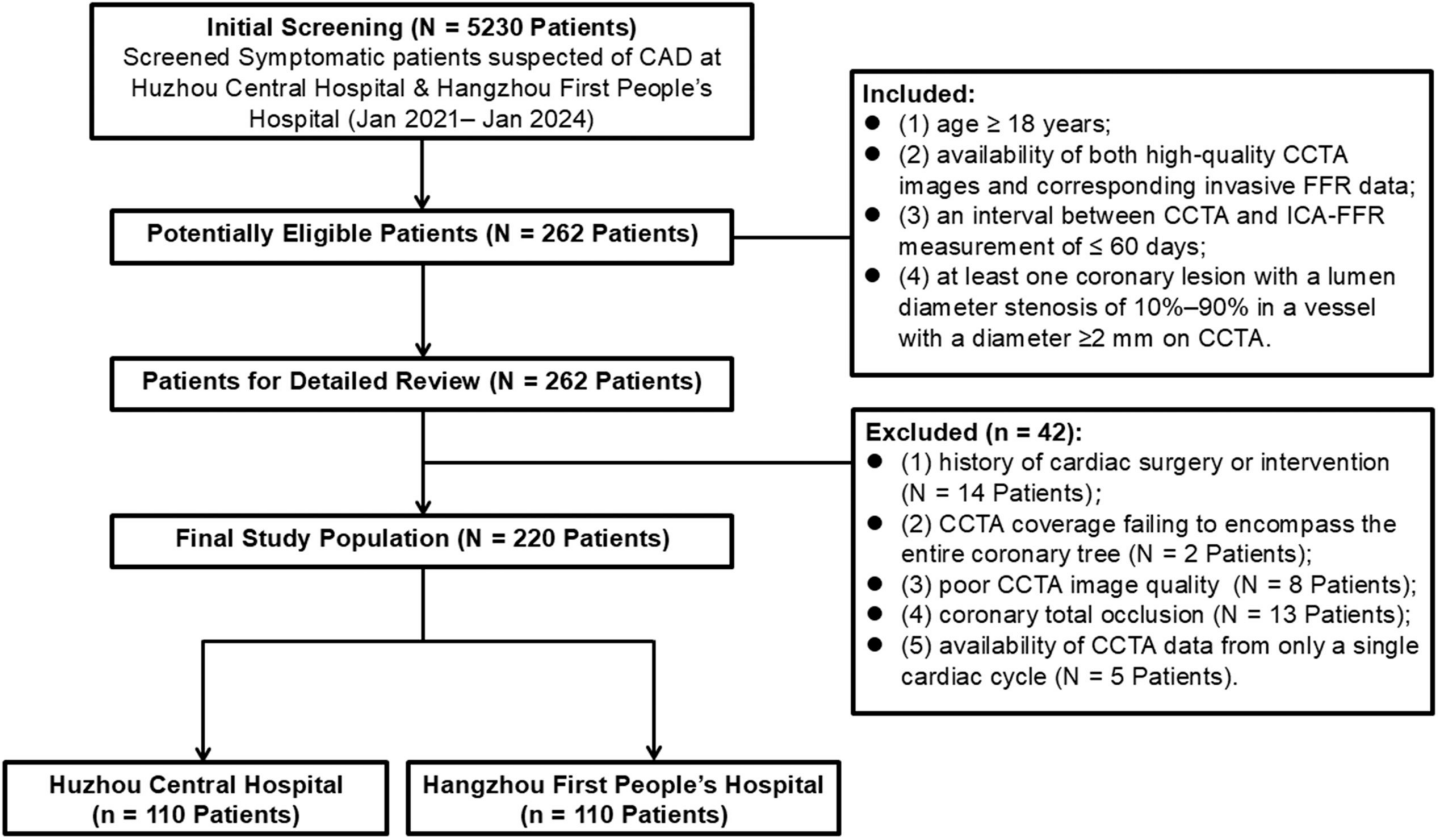
Study flowchart of patient enrollment and analysis.

This retrospective, multi-center study consecutively screened symptomatic patients suspected of CAD at Huzhou Central Hospital and Hangzhou First People's Hospital between January 2021 and January 2024. A total of 220 patients (277 vessels), with 110 patients from each center, were finally enrolled based on predefined inclusion and exclusion criteria (Figure 1).

The inclusion criteria were as follows: (1) age ≥ 18 years; (2) availability of both high-quality CCTA images and corresponding invasive FFR data; (3) an interval between CCTA and ICA-FFR measurement of ≤ 60 days; and (4) at least one coronary lesion with a lumen diameter stenosis of 10%–90% in a vessel with a diameter ≥2 mm on CCTA. Patients were excluded if they met any of the following criteria: (1) history of cardiac surgery or intervention, including coronary artery bypass grafting, percutaneous coronary intervention, prosthetic valve replacement, or implantation of a pacemaker/ICD; (2) CCTA coverage failing to encompass the entire coronary tree; (3) poor CCTA image quality due to severe motion, step-section, beam-hardening, or metallic artifacts; (4) coronary total occlusion; or (5) availability of CCTA data from only a single cardiac cycle.

Clinical characteristics were retrieved from electronic medical record systems, and imaging data were exported from Picture Archiving and Communication Systems. To ensure data consistency between the two medical centers, all parameters were recorded in a standardized format and managed in a centralized database. This retrospective study was approved by the Ethics Committee of the HuZhou Central Hospital (Approval No.2025-206-02), and the requirement for informed consent was waived due to the retrospective nature of the study.

### CCTA acquisition and image reconstruction

All CCTA examinations were performed using third-generation or second-generation dual-source CT scanners (Somatom Force or Somatom Definition Flash, Siemens Healthineers, Forchheim, Germany). To ensure standardization across both centers, scan protocols were strictly aligned with the Society of Cardiovascular Computed Tomography guidelines (19).

Patients were instructed to rest for at least 30 minutes prior to the scan. Venous access was established via the right median cubital vein using an 18G cannula (BD Intima II). For patients with a resting heart rate >75 bpm and no contraindications, oral β-blockers (metoprolol, 25–75 mg; AstraZeneca) were administered before CCTA. All patients without contraindications received 0.5 mg sublingual nitroglycerin (Yimin Pharmaceutical) one minute before scanning to optimize coronary vasodilation.

A non-contrast scan was first performed with prospective ECG-triggering at 70% of the R-R interval to calculate the Agatston coronary artery calcium score with a 3 mm slice thickness. Then, CCTA images were acquired with prospective ECG-triggering over the 35%-75% R-R interval. We used a bolus-tracking technique, and the region of interest was placed in the descending aorta. Scanning started automatically 6 seconds after attenuation reached 100 HU. The scan settings included a tube voltage of 70-120 kV and a reference tube current of 346 mAs (CareDose 4D). Contrast medium (Ioversol, 350 mgI/mL, Hengrui Medicine) was injected at 4.0-5.5 mL/s, and then a 40 mL saline flush was given. Images were reconstructed with a slice thickness of 0.75 mm and an increment of 0.6 mm. The system automatically selected the best systolic and diastolic phases, and manual adjustment was made when needed to get the best image quality. All DICOM data were sent to a dedicated workstation (Syngo.via VB10/20, Siemens Healthineers) for standard post-processing.

### ICA and invasive FFR measurement

**Figure 2:**
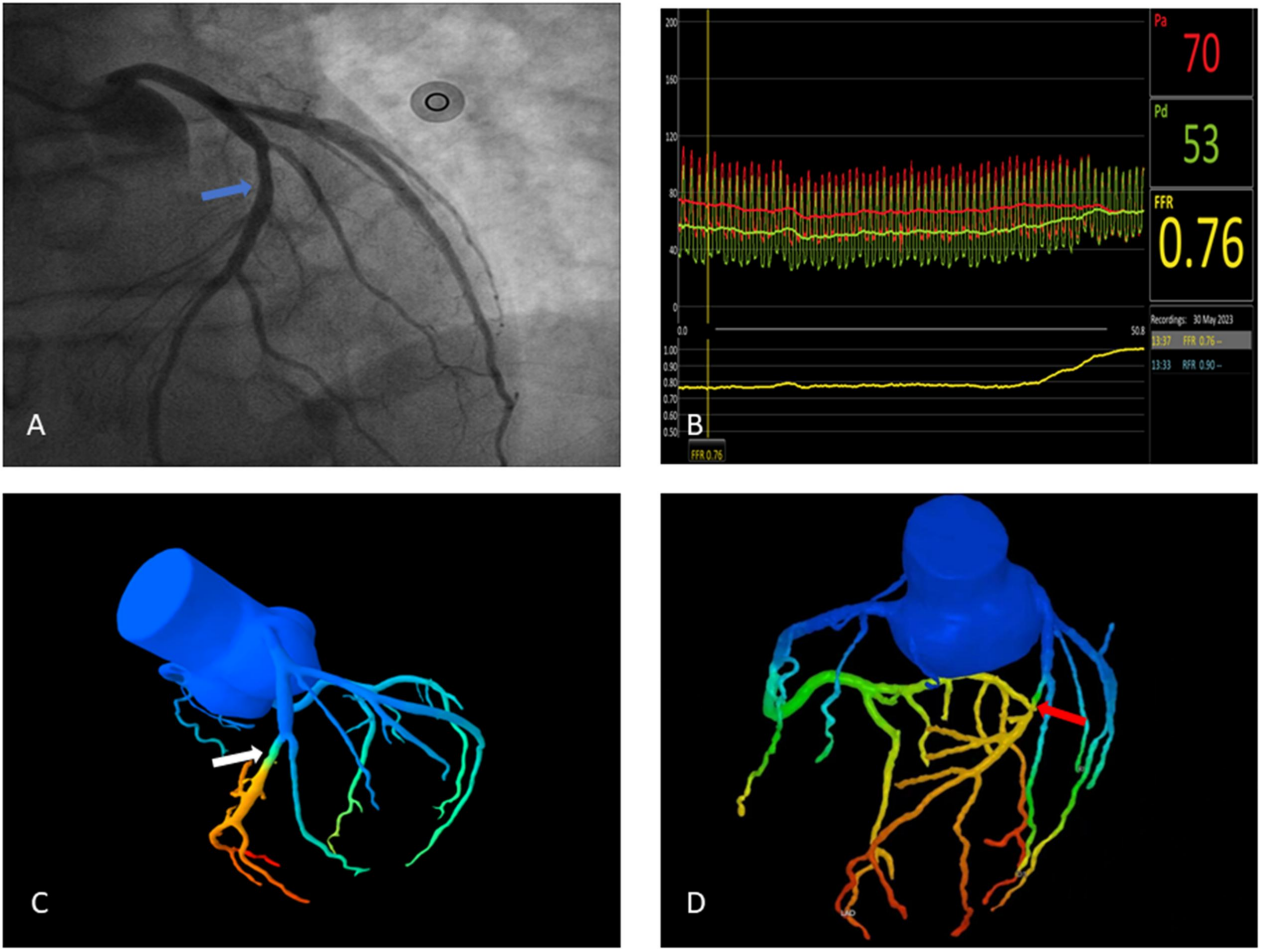
Representative case of a 53-year-old male with stable chest pain. **(A)** Invasive coronary angiography (ICA) showing a significant stenosis in the mid-left anterior descending artery (LAD; blue arrow). **(B)** Invasive FFR measurement confirming functional ischemia with a value of 0.76. **(C)** DL-based CT-FFR color-coded map showing a comparable result (CT-FFR = 0.75; white arrow). **(D)** CFD-based CT-FFR color-coded map demonstrating diagnostic consistency (CT-FFR = 0.74; red arrow).

ICA and FFR measurements were performed according to standard clinical guidelines (20). Selective catheterization was done through transradial or transfemoral access, and at least three near-orthogonal projections were obtained for each major artery to get clear visualization (Figure 2A). After ICA, invasive FFR was measured for lesions with suspected functional significance using a 0.014-inch pressure wire (St. Jude Medical, Minneapolis, MN, USA). After the pressure between the wire sensor and the guiding catheter was equalized at the coronary ostium, the wire was advanced 1-2 cm distal to the target lesion. Maximal hyperemia was induced by continuous intravenous infusion of adenosine at a dose of 140-180?g/kg/min for at least 2 minutes. FFR? 0.80 was defined as hemodynamically significant ischemia. All procedures were stored in the institutional PACS for later correlation analysis with CT-FFR.

### CT-FFR Algorithms

CT-FFR analyses were performed using two representative commercially available algorithms by operators blinded to invasive FFR results: a CFD-based approach (Ruixin-FFR, Ruixin Medical) and a DL-based approach (Shukun CT-FFR, Shukun Technology). Because both platforms are proprietary commercial systems, source code, model weights, exact training datasets, solver implementation details, and some numerical parameters are not publicly disclosed. To improve methodological transparency, the following description summarizes the publicly available technical principles reported for these platforms and the information available from the vendor workflow (6, 9, 10, 21).

1. CFD-based Algorithm: The Ruixin-FFR method combines AI-assisted coronary reconstruction with CFD simulation. According to the published description of this algorithm, three-dimensional coronary tree models are reconstructed from CCTA through four automated steps: coarse coronary tree segmentation using a deep-learning method and largest connected-component detection, automatic centerline extraction by region growing, lumen contour refinement using a Coarse-to-Fine Subpixel algorithm, and coronary surface reconstruction by spline interpolation and lofting of cross-sectional contours (21). The coronary arterial model is then automatically divided into unstructured grids for CFD analysis. Patient-level left ventricular myocardial volume is derived from CCTA, resting total coronary flow is estimated using modified allometric scaling laws, flow is distributed according to branch number and outlet area, and maximal hyperemia is simulated by scaling coronary flow/resistance parameters (21). The three-dimensional, steady-state, incompressible Navier-Stokes equations are solved using a finite element method to obtain velocity and pressure fields. CT-FFR is calculated from the simulated pressure ratio (Pd/Pa), with lesion-specific values extracted at the site corresponding to invasive FFR measurement or distal to the target stenosis. Detailed mesh density, mesh-independence thresholds, numerical scheme settings, convergence criteria, and proprietary boundary-condition tuning are not disclosed by the commercial software vendor.
2. DL-based Algorithm: The Shukun CT-FFR platform is an artificial intelligence-based fully automated on-site CT-FFR system. Publicly available technical reports describe two major steps: automatic coronary artery reconstruction and CT-FFR calculation (6, 10). Coronary reconstruction is performed using a modified U-net architecture with bottleneck blocks and transformer blocks with multi-head self-attention; in the reported development work, 2144 well-labeled CCTA scans from six Chinese medical centers were used to train the coronary reconstruction model (10). Plaque detection and lumen extraction are further supported by a coronary-structure-guided 3D + 2D convolutional neural network with adaptive receptive fields and multi-head self-attention, enabling automatic reconstruction of the coronary tree including branch vessels of approximately 1-2 mm in diameter(10). The CT-FFR calculation uses the reconstructed coronary geometry and personalized physiological parameters; published descriptions report patient-specific inlet pressure when available, outlet flow resistance assignment, Murray law-based flow distribution, and hyperemic resistance reduction (10). The software outputs a color-coded CT-FFR map and lesion-level CT-FFR values. The exact FFR prediction architecture, training labels for the final CT-FFR estimation module, model weights, overfitting-control procedures, and full external validation datasets are proprietary and are not fully disclosed in the public literature. No fine-tuning or recalibration was performed using the present study cohort.

### Statistical analysis

Statistical analyses were performed using SPSS version 26.0. Continuous variables were expressed as mean ± standard deviation (SD) or median (interquartile range, IQR), depending on the normality of the distribution, which was assessed using the Shapiro–Wilk test. Categorical variables were compared using the chi-square test or Fisher's exact test, as appropriate. The diagnostic performance of DL-based and CFD-based CT-FFR was evaluated using invasive FFR ≤ 0.80 as the reference standard. Key diagnostic metrics, including sensitivity, specificity, positive predictive value (PPV), negative predictive value (NPV), and accuracy, were calculated. Receiver operating characteristic (ROC) curves were constructed, and the area under the curve (AUC) was calculated to assess the overall diagnostic efficacy. The DeLong test was employed to compare the differences in AUC between the two algorithms and across various subgroups. In ischemia-positive gray-zone lesions, defined as invasive FFR values from 0.75 to 0.80, CT-FFR ≤ 0.80 was considered correct classification, whereas CT-FFR > 0.80 was considered false-negative classification. Correct classification rates were compared using McNemar's test, and continuous gray-zone metrics were compared using paired tests. To evaluate the consistency and correlation between CT-FFR and invasive FFR, Bland-Altman analysis and Spearman's (or Pearson's) correlation coefficients were utilized. A two-tailed *p* value < 0.05 was considered statistically significant.

## Results

### Patient characteristics

A total of 220 patients (277 vessels) from two clinical centers were included in the final analysis. Based on invasive FFR, which was the gold standard, patients were divided into the ischemia-positive group (*n* = 186) and the ischemia-negative group (*n* = 34). As shown in Table 1, there were no significant differences between the two groups in most demographic data and clinical history, including age, sex, and BMI (all *p* > 0.05). For cardiovascular risk factors, the rates of diabetes, hyperlipidemia, heart failure, and stroke were similar between groups (*p* > 0.05), but smoking was more common in the ischemia-positive group (*p* < 0.05). Imaging-related measures, including pericoronary adipose tissue attenuation and Agatston coronary artery calcium score, also showed no significant difference. So, except for smoking history, the baseline clinical and imaging features were similar in the two groups, which provided a solid basis for evaluating the diagnostic performance of the CT-FFR algorithms.

**Table 1:** Baseline clinical and imaging characteristics of the study population.

Variables	FFR ≤ 0.80 (*n* = 186)	FFR > 0.80 (*n* = 34)	t/Z/*χ*^2^	P
Age (years)	65.72 ± 9.57	66.65 ± 9.86	−0.52	0.604
Sex			1.813	0.178
Female	65 (34.9%)	16 (47.1%)		
Male	121 (65.1%)	18 (52.9%)		
BMI	24.36 ± 2.94	24.06 ± 3.52	0.537	0.592
Smoking history			4.034	0.045
No	127 (68.3%)	29 (85.3%)		
Yes	59 (31.7%)	5 (14.7%)		
History of CAD			0.398	0.528
No	93 (50.0%)	15 (44.1%)		
Yes	93 (50.0%)	19 (55.9%)		
History of Hypertension			0.416	0.519
No	71 (38.2%)	11 (32.4%)		
Yes	115 (61.8%)	23 (67.6%)		
History of Diabetes mellitus			1.061	0.303
No	144 (77.8%)	29 (85.3%)		
Yes	42 (22.2%)	5 (14.7%)		
History of Hyperlipidemia			0.596	0.440
No	167 (89.8%)	29 (85.3%)		
Yes	19 (10.2%)	5 (14.7%)		
History of Heart failure			0.239	0.625
No	177 (95.2%)	33 (97.1%)		
Yes	9 (4.8%)	1 (2.9%)		
History of stroke			0.556	0.456
No	183 (98.4%)	34 (100.0%)		
Yes	3 (1.6%)	0 (0.0%)		
Pericoronary adipose tissue attenuation (HU)	−87.09 (−111.65, −76.42)	−89.70 (−107.55, −76.72)	0.158	0.874
Coronary artery calcium score	2.86 (0.00, 65.00)	2.70 (0.00, 41.67)	0.465	0.642

### Overall diagnostic performance of DL-based and CFD-based CT-FFR

**Figure 3:**
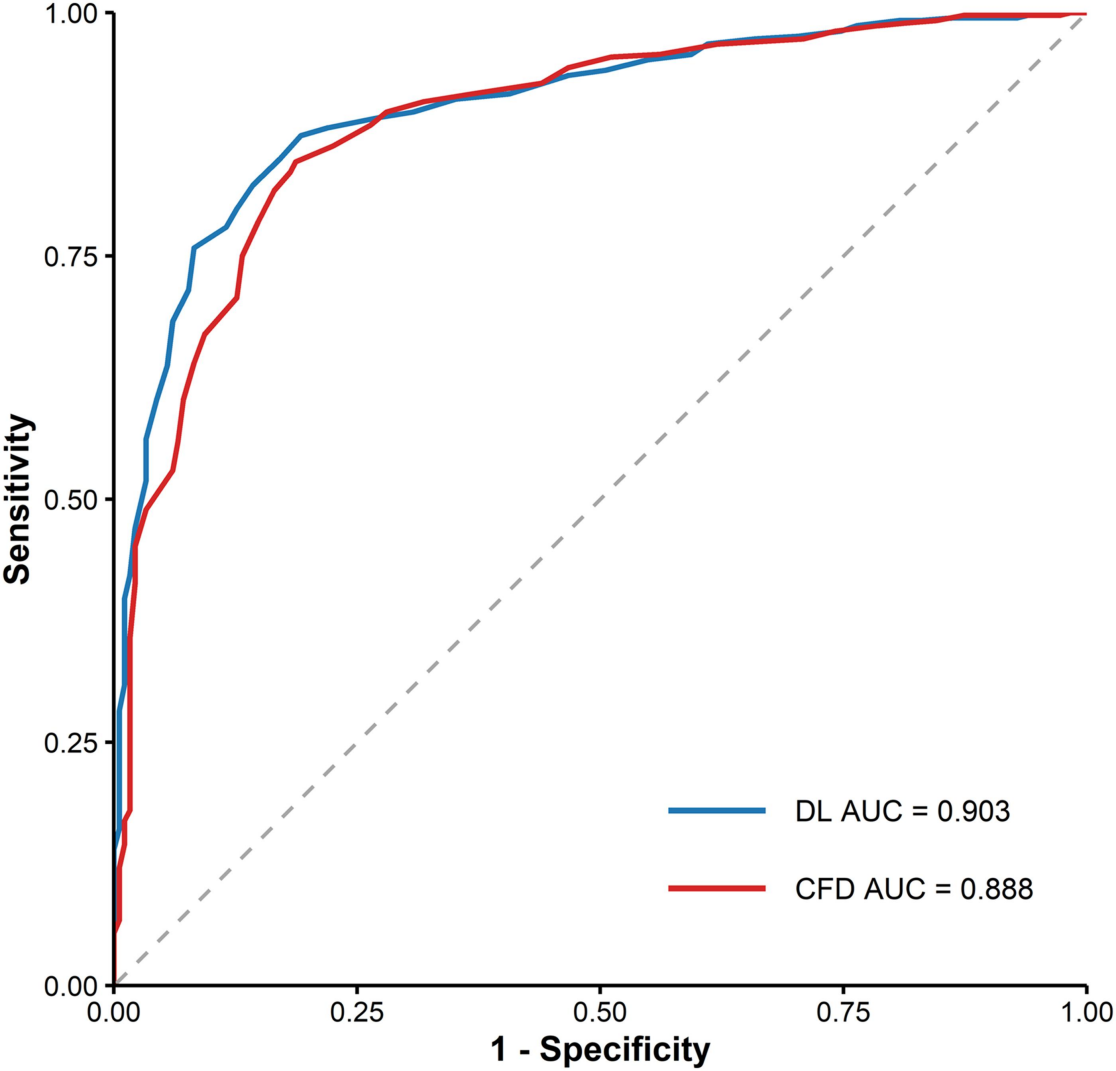
Receiver operating characteristic curves for overall diagnostic performance.

Table 2 shows the overall diagnostic performance of the DL-based and CFD-based CT-FFR algorithms, with invasive FFR as the gold standard. Both algorithms showed high diagnostic value for detecting hemodynamically significant coronary stenosis. The ROC AUC was 0.90 (0.88-0.93) for DL-based CT-FFR and 0.89 (0.86-0.92) for CFD-based CT-FFR (Figure 3). The DeLong test showed no significant difference between the two AUCs (*p* = 0.33), so the two algorithms had similar diagnostic power.

**Table 2:** Overall diagnostic performance of DL-based and CFD-based CT-FFR algorithms.

Metric	DL-based CT-FFR	CFD-based CT-FFR
AUC (95% CI)	0.903 (0.877-0.929)	0.888 (0.859-0.917)
Sensitivity	91.7%	91.7%
Specificity	59.3%	63.2%
PPV	82.2%	83.6%
NPV	77.7%	78.8%
Accuracy	81.0%	82.3%

**Figure 4:**
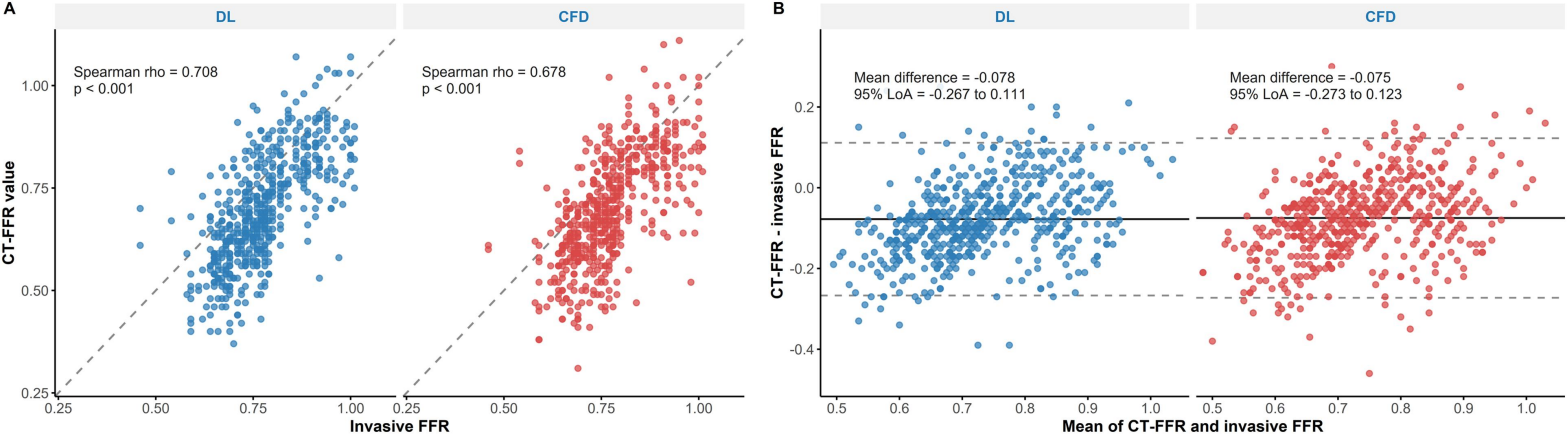
Correlation and consistency analysis of CT-FFR vs. Invasive FFR. **(A)** Spearman correlation plots for DL-based and CFD-based CT-FFR compared with invasive FFR. **(B)** Bland-Altman plots showing mean bias and 95% limits of agreement for each algorithm.

For DL-based and CFD-based CT-FFR, the sensitivity was 91.7% and 91.7%, the specificity was 59.3% and 63.2%, the PPV was 82.2% and 83.6%, and the NPV was 77.7% and 78.8%, respectively. Both CT-FFR algorithms were strongly correlated with invasive FFR, with Spearman's rho values of 0.71 for DL-based CT-FFR and 0.68 for CFD-based CT-FFR (both *p* < 0.001; Figure 4A). Bland-Altman analysis also showed good agreement between the two non-invasive methods and the gold standard, and most data points were within the 95% limits of agreement (Figure 4B).

### Subgroup stability analysis

To assess the stability of both CT-FFR algorithms in different clinical and technical settings, we performed subgroup analyses by target vessel (LAD, LCX, and RCA), cardiac phase (diastolic vs. systolic), and study center (Center 1 vs. Center 2).

**Figure 5:**
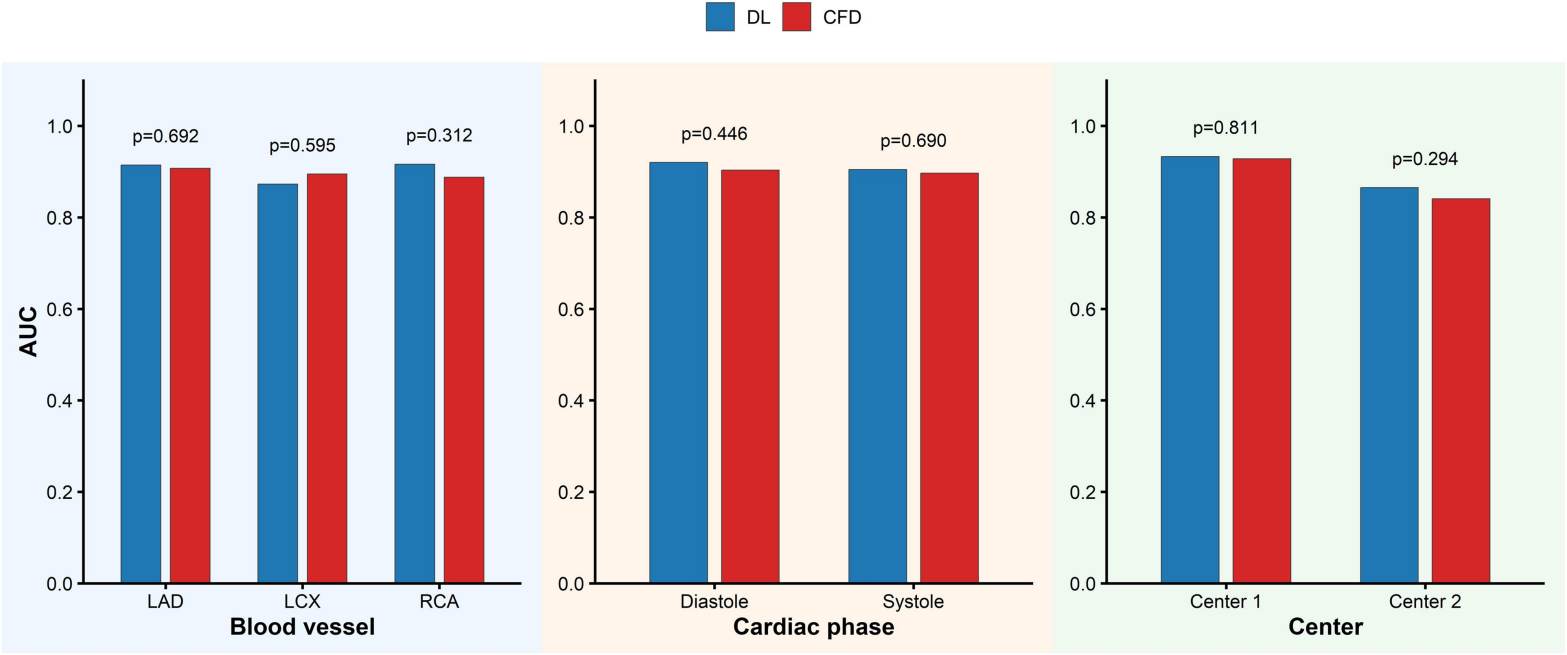
Subgroup analysis of diagnostic performance across clinical and technical scenarios.

As shown in Figure 5, both DL-based and CFD-based algorithms kept stable diagnostic performance in all subgroups. In the vessel-specific analysis, the AUCs for LAD, LCX, and RCA were 0.91 (0.88,0.95), 0.87 (0.81,0.93), and 0.92 (0.87,0.97) for the DL algorithm, and 0.91 (0.87,0.95), 0.89 (0.84,0.95), and 0.89 (0.83,0.94) for the CFD algorithm. There was no significant difference among the three major coronary branches (*p* > 0.05). For cardiac phase, both algorithms showed similar results in the systolic and diastolic phases, with AUCs of 0.90 (0.87,0.94) vs. 0.92 (0.89,0.95) for DL and 0.90 (0.86,0.93) vs. 0.90 (0.86,0.94) for CFD (*p* > 0.05). Center-based validation also showed stable diagnostic accuracy in Center 1 and Center 2 (AUC: 0.93 (0.91,0.96) vs. 0.87 (0.82,0.91) for DL; 0.93 (0.89,0.96) vs. 0.85 (0.79,0.89) for CFD; all *p* > 0.05). These results show that both CT-FFR methods were reliable and were not clearly affected by vessel location, reconstruction phase, or center-related technical variation.

### Diagnostic performance in the clinical “gray zone”

The clinical gray zone was defined as ischemia-positive borderline lesions with invasive FFR values from 0.75 to 0.80. In this study, 162 vessels were included in the gray-zone analysis. Correct classification was defined as CT-FFR ≤ 0.80, whereas CT-FFR > 0.80 was considered false-negative classification. As shown in Table 3, DL-based CT-FFR correctly classified 140 vessels (86.4%), whereas CFD-based CT-FFR correctly classified 137 vessels (84.6%; *p* = 0.690). The corresponding false-negative rates were 13.6% and 15.4%, respectively. The mean CT-FFR values were similar between DL and CFD (0.686 ± 0.097 vs. 0.691 ± 0.103; *p* = 0.500). The bias versus invasive FFR was −0.088 ± 0.095 for DL and −0.083 ± 0.101 for CFD (*p* = 0.500), and the mean absolute error was also comparable between the two algorithms (0.112 ± 0.065 vs. 0.107 ± 0.075; *p* = 0.432). These findings suggest that DL-based and CFD-based CT-FFR had comparable detection performance in ischemia-positive gray-zone lesions.

**Table 3:** Detection performance of CT-FFR algorithms in ischemia-positive clinical gray-zone lesions.

Metric	DL-based CT-FFR	CFD-based CT-FFR	P
Gray-zone vessels, n	162	162	-
Mean invasive FFR	0.774 ± 0.016	0.774 ± 0.016	-
Mean CT-FFR value	0.686 ± 0.097	0.691 ± 0.103	0.500
Correctly classified vessels, n (%)	140 (86.4%)	137 (84.6%)	0.690
False-negative vessels, n (%)	22 (13.6%)	25 (15.4%)	0.690
Sensitivity	86.4%	84.6%	0.690
Mean absolute error	0.112 ± 0.065	0.107 ± 0.075	0.432
Bias vs invasive FFR	−0.088 ± 0.095	−0.083 ± 0.101	0.500

## Discussion

In this multi-center head-to-head study, we compared the diagnostic performance and stability of two representative CT-FFR strategies, DL-based and CFD-based, using invasive FFR as the reference standard in the same patient and vessel cohort. The principal finding was that both methods achieved high and similar diagnostic performance for identifying hemodynamically significant coronary stenosis. Importantly, their performance remained stable across major coronary arteries, systolic and diastolic reconstruction phases, and two imaging centers. In ischemia-positive clinical gray-zone lesions, both algorithms showed comparable correct classification rates, false-negative rates, bias, and mean absolute error. These findings extend previous single-algorithm validation studies by showing that two different commercial computational approaches can provide similar functional information when applied to the same multi-center CCTA dataset.

The diagnostic accuracy observed in our study is consistent with the broader CT-FFR literature. The NXT trial established the clinical value of CFD-based CT-FFR and reported an AUC of 0.90 for identifying lesion-specific ischemia (4). Recent multicenter and real-world studies have further shown that CT-FFR can improve functional assessment beyond CCTA anatomy alone and may reduce unnecessary invasive coronary angiography without compromising short-term clinical outcomes (5, 6, 22–24). For AI- or DL-enabled CT-FFR, published studies have reported encouraging diagnostic and prognostic performance, faster on-site processing, and high technical success rates (10–14). Our results are aligned with these reports and add a direct same-cohort comparison between DL-based and CFD-based CT-FFR. A recent external comparison of AI-based and CFD-based FFR software in intermediate-grade stenosis also found value in testing these approaches against invasive physiologic assessment, supporting the importance of comparative evaluation rather than single-algorithm validation alone (15).

The similarity between DL-based and CFD-based CT-FFR requires cautious interpretation. One possible explanation is that both algorithms depend strongly on the same initial information: CCTA-derived coronary lumen geometry, centerline quality, lesion location, and image quality. When segmentation and anatomical reconstruction are reliable, both a physics-based pressure-flow simulation and a data-driven model trained to approximate hemodynamic behavior may converge toward similar lesion-level FFR estimates. In addition, modern AI-based CT-FFR systems may incorporate physiologic rules, reduced-order hemodynamic models, or training references derived from invasive FFR and/or CFD simulation, which could further reduce apparent differences between methods (10, 14). Therefore, our findings should not be interpreted as proof that CT-FFR is completely algorithm-independent. Rather, they suggest that, under standardized acquisition conditions and in a selected cohort without severely non-diagnostic images or very complex anatomy, both computational strategies can produce comparable clinically useful information.

The stability of CT-FFR algorithms in ischemia-positive gray-zone lesions is clinically important. Previous work has shown that CT-FFR performance may decrease near the physiological decision threshold, where small changes in lumen segmentation, calcium blooming, image noise, boundary-condition assumptions, or model estimation can influence binary classification (17, 18). Despite this challenge, both algorithms in our study showed comparable correct classification rates and false-negative rates in gray-zone lesions. The two algorithms also showed similar bias and mean absolute error versus invasive FFR, suggesting similar performance for borderline ischemia-positive disease. In practice, however, CT-FFR values close to 0.80 should still be interpreted together with symptoms, stenosis morphology, plaque burden, image quality, and other clinical information rather than used as an isolated binary result. Recent comprehensive methodology reviews, literature appraisals, and regional status updates similarly emphasize that clinical translation should balance the advantages of non-invasive functional assessment with attention to algorithm transparency, vendor-specific assumptions, image-quality dependence, and validation in challenging real-world subgroups (9, 16, 25, 26).

This study has several limitations. First, the retrospective design may have introduced selection bias. Although patients were consecutively screened from two centers to reduce this risk, prospective multicenter studies with larger sample sizes are needed to validate these findings in broader clinical settings. Second, although the sample size of 220 patients was sufficient for the primary comparison between the two algorithms, it remains relatively modest compared with large international multicenter registries. A larger cohort would allow more detailed subgroup analyses, particularly in high-risk populations. Third, patients with complex coronary anatomy, including chronic total occlusion, prior coronary artery bypass grafting, and severe calcification, were excluded. Therefore, the performance of DL-based and CFD-based CT-FFR in these challenging lesions requires further investigation. Fourth, both CT-FFR platforms are commercial software systems. Although we expanded the Methods section using publicly available technical literature, the exact source code, model weights, full training datasets, numerical solver settings, convergence criteria, and mesh-independence procedures were not available to the authors because they are proprietary. This limits independent reproducibility of some technical components and should be considered when interpreting the head-to-head comparison. Fifth, this study mainly evaluated per-vessel and per-patient diagnostic performance, without assessing long-term clinical outcomes or cost-effectiveness. Future studies incorporating follow-up data are warranted to determine the real-world impact of these technologies on patient management and healthcare costs.

## Conclusion

In conclusion, this multi-center head-to-head study shows that DL-based and CFD-based CT-FFR algorithms have high and similar diagnostic performance for identifying hemodynamically significant coronary artery disease. Both methods showed stable performance across different coronary branches, cardiac phases, and imaging centers, and comparable detection performance in ischemia-positive clinical gray-zone lesions.

## Data Availability

The raw data supporting the conclusions of this article will be made available by the authors, without undue reservation.
